# Neuroticism polygenic risk predicts conversion from mild cognitive impairment to Alzheimer's disease by impairing inferior parietal surface area

**DOI:** 10.1002/hbm.26709

**Published:** 2024-05-15

**Authors:** Qiaojun Li, Xingping Lv, Qian Qian, Kun Liao, Xin Du, for the Alzheimer's Disease Neuroimaging Initiative

**Affiliations:** ^1^ College of Information Engineering Tianjin University of Commerce Tianjin China; ^2^ College of Sciences Tianjin University of Commerce Tianjin China; ^3^ Department of Radiology and Tianjin Key Laboratory of Functional Imaging Tianjin Medical University General Hospital Tianjin China

**Keywords:** Alzheimer's disease, amnestic mild cognitive impairment, inferior parietal surface area, neuroticism, polygenic risk score

## Abstract

The high prevalence of conversion from amnestic mild cognitive impairment (aMCI) to Alzheimer's disease (AD) makes early prevention of AD extremely critical. Neuroticism, a heritable personality trait associated with mental health, has been considered a risk factor for conversion from aMCI to AD. However, whether the neuroticism genetic risk could predict the conversion of aMCI and its underlying neural mechanisms is unclear. Neuroticism polygenic risk score (N‐PRS) was calculated in 278 aMCI patients with qualified genomic and neuroimaging data from ADNI. After 1‐year follow‐up, N‐PRS in patients of aMCI‐converted group was significantly greater than those in aMCI‐stable group. Logistic and Cox survival regression revealed that N‐PRS could significantly predict the early‐stage conversion risk from aMCI to AD. These results were well replicated in an internal dataset and an independent external dataset of 933 aMCI patients from the UK Biobank. One sample Mendelian randomization analyses confirmed a potentially causal association from higher N‐PRS to lower inferior parietal surface area to higher conversion risk of aMCI patients. These analyses indicated that neuroticism genetic risk may increase the conversion risk from aMCI to AD by impairing the inferior parietal structure.

## Graphical abstract

This study developed a neurotic polygenic risk score (N‐PRS) that predicted progression from amnestic mild cognitive impairment (aMCI) to Alzheimer's disease (AD) and explored its potential causal relationship with brain structure.

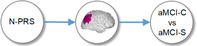

## 1 INTRODUCTION

The amnestic mild cognitive impairment (aMCI) is a symptomatic predementia stage of Alzheimer's disease (AD), with heterogeneous genetic risk that gradually impairs advanced cognitive ability (Portet et al., 2006). The aMCI patients have a high risk of converting to AD within 5 years (Association, 2023), thus, early prediction of aMCI conversion is particularly important for mitigating AD onset and stratifying clinical populations (Chaudhury et al., 2019; Zhou et al., 2021). Multiple demographic (Mitchell & Shiri‐Feshki, 2009; Ward et al., 2013), clinical (Agostini et al., 2016; Tarawneh & Holtzman, 2012), psychological (Mazzeo et al., 2016; Perri et al., 2007), and neuroimaging biomarkers (Spalletta et al., 2014; Yuan et al., 2009) have been considered as potential risk factors that contributed to the conversion of aMCI to AD at early stage. Among these factors, neuroticism, a personality trait, reflecting emotional instability of negative emotions, poor self‐regulation, trouble dealing with stress, and prone to complain (Widiger & Oltmanns, 2017), were preliminarily found to predict the conversion from aMCI to AD (Li et al., 2021; Segerstrom, 2020; Terracciano et al., 2022).

Twin genetic studies have shown that heritability of neuroticism was 41%–48% (Jang et al., 1996; van den Berg et al., 2014). Genome‐wide association studies (GWAS) have identified statistically 136 independent genetic loci associated with neuroticism in a large sample of 449,484 participants (Nagel et al., 2018). Rather than using genetic variants that reach statistically genome‐wide significance, polygenic risk score (PRS) was calculated by weighting the risk genetic allele numbers with the corresponding effect size estimated from the summary statistics of GWAS (Purcell et al., 2009). PRS could explain more variations for complex traits that would hardly be detected using a single genetic variant with a small effect size. The PRS for depression has been found to predict the aMCI conversion (Xu et al., 2018). The neuroticism, as a pervasive risk factor and important endophenotype for depression, whether the PRS for neuroticism (N‐PRS) could predict the conversion from aMCI to AD and its underlying neural mechanisms were still unknown.

In this study, we calculated the N‐PRS to assess the cumulative neuroticism genetic risk in 278 aMCI patients from the Alzheimer's Disease Neuroimaging Initiative (ADNI) and 933 aMCI patients from the UK Biobank (UKBB) (Sudlow et al., 2015). First, in 278 aMCI patients from ADNI, we evaluated and replicated the predictive effect of the N‐PRS on the conversion from aMCI to AD at 1‐ and 10‐year follow‐up. Second, in 933 aMCI patients from UKBB, we additionally tested the repeatability of the prediction effect of the N‐PRS on the conversion of aMCI. If so, we would like to further explore the neural mechanism underlying the prediction effect of the N‐PRS on aMCI conversion. Using N‐PRS as an instrumental variable (IV), there were two ways of brain structural changes underlying aMCI conversion: one is that the brain structural changes contributed to aMCI conversion and the other is that aMCI conversion results in subsequent brain structural impairment. Only the former potentially causal association could demonstrate the neural mechanism underlying the prediction effect of the N‐PRS on the aMCI conversion. Thus, using one sample Mendelian randomization analyses, we finally investigated the potentially causal association between the N‐PRS, brain structure, and aMCI conversion.

## 2 MATERIALS AND METHODS

### 2.1 Analyses sample

#### 2.1.1 Alzheimer's Disease Neuroimaging Initiative

In this study, N‐PRS was used to predict the conversion from aMCI to AD. Calculating the N‐PRS required a discovery sample and a target sample. The discovery sample was used to identify the effect size of a set of genetic variants that were nominally associated with neuroticism at a predefined *p* value. Then, the N‐PRS was calculated to estimate the cumulative genetic risk of neuroticism for each aMCI patient in the target sample of ADNI. To test the repeatability of the prediction effect for aMCI conversion, we used two discovery samples to construct N‐PRS in aMCI patients from ADNI separately. We summarized the discovery and target samples here.

*Discovery sample one*. The largest and most powerful GWAS of neuroticism was used as the first discovery sample (Nagel et al., 2018). The GWAS summary statistics data was from a meta‐analysis of GWAS for neuroticism in 449,484 individuals. The neuroticism meta‐analysis comprised data from the UKBB (*n* = 372,903), 23andMe (*n* = 59,206) and the first stage Genetics of Personality Consortium (GPC‐1) (*n* = 17,375). The detailed information was shown in the original work (Nagel et al., 2018).

*Discovery sample two*. To exclude the technical bias of the first discovery neuroticism GWAS sample, we constructed another N‐PRS based on the second discovery sample to replicate the prediction effect of N‐PRS on aMCI conversion in ADNI. GWAS summary statistics data of GWAS for neuroticism from the second stage GPC (GPC‐2) was used as the second discovery sample (Genetics of Personality Consortium et al., 2015; van den Berg et al., 2014) (https://tweelingenregister.vu.nl/gpc). GPC‐2 is a large GWAS project of personality within 30 study groups from the United States, Europe, and Australia, including a total of 73,447 participants. Neuroticism personality in GPC‐2 was assessed using the Item Response Theory.

*Target sample*. We used the first stage of the ADNI (ADNI‐1) dataset as our target sample (Mueller et al., 2005; Weiner et al., 2010) (http://www.adni-info.org). Participants were carefully screened for drug or alcohol abuse and had no contraindications to MRI. Detailed inclusion and exclusion criteria can be found in the ADNI protocol (Mueller et al., 2005). At baseline, among 798 participants from ADNI‐1, there are 200 normal controls, 398 aMCI patients, and 200 AD patients. Our sample was screened from 398 aMCI patients diagnosed with ADNI‐1. All aMCI participants were diagnosed with objective memory impairment by Petersen et al. criteria and had no significant impairment in other cognitive domains. The detailed sample screening process is shown in Table S1. Finally, we used 304 aMCI patients with qualified genetic and follow‐up diagnosis data (201 males; mean and standard deviation age at baseline: 75.02 ± 7.20 years). The aMCI patients in ADNI‐1 had been followed up to determine whether converted to AD (aMCI‐C) or not (aMCI‐S) after 1‐ and 10‐year follow‐up, separately. Thus, N‐PRS_1_ demonstrated cumulative genetic risk for neuroticism based on the first discovery sample in the target ADNI sample, N‐PRS_2_ demonstrated cumulative genetic risk for neuroticism based on the second discovery sample in the target ADNI sample.

#### 2.1.2 UK Biobank

*Discovery sample*. As the first discovery sample of neuroticism GWAS summary data (Nagel et al., 2018) included 372,903 participants from UKBB, where PRS results can be substantially inflated even in the presence of minimal sample overlap between the discovery and target sample (Wray et al., 2013). Thus, we selected GWAS summary statistics data for neuroticism from GPC‐2 (not including the UKBB cohort) as the discovery sample (Genetics of Personality Consortium et al., 2015; van den Berg et al., 2014) to generate N‐PRS in the target UKBB sample.

*Target sample*. The target sample used in this study were from the UKBB (www.ukbiobank.ac.uk) (Sudlow et al., 2015). The main goals of UKBB are to explore the etiology of common complex diseases by investigating their association with underlying genetic and lifestyle determinants, which may contribute to the advancement of modern medicine and treatment that improve human health. Informed consent was obtained from all UKBB participants. To validate the prediction effect of N‐PRS in aMCI patients from the ADNI, we created a subset dataset including 1065 aMCI patients from UKBB. Detailed information about this UKBB subset is in Supporting Information Materials and Methods. Finally, after quality control, 933 aMCI patients were finally used (516 males; mean and standard deviation age at baseline: 62.77 ± 6.03 years).

### 2.2 Genetic data process

#### 2.2.1 Alzheimer's Disease Neuroimaging Initiative

##### Genotyping

The genome‐wide genetic variants from ADNI‐1 were genotyped using the Illumina Human610‐Quad Bead chip (https://ida.loni.usc.edu). Detailed information on the genetic microarray was shown in the ADNI protocol.

##### Quality control

Quality control of genetic data was performed by PLINK v2.0 (http://www.cog-genomics.org/plink2/) (Chang et al., 2015; Purcell et al., 2007). Detailed information on quality control and imputation were shown in the previous work (Xu et al., 2018). We summarized the process here:

*SNP‐level quality control*. In the SNP‐level quality control, we excluded the Genetic variants with missing call rate higher than 0.05, minor allele frequency (MAF) less than 0.01, severe deviation from Hardy–Weinberg equilibrium (*p* < .5 × 10^−6^) and ambiguous strand.

*Sample‐level quality control*. In the sample‐level quality control, we excluded participants with gender mismatch between self‐reported and genotyping data, close relations estimated by identity by descent (IBD > 0.1875), excess heterozygosity (>mean ± 5 SD), missing genotypes >3%, and outliers relative to EAS identified by principal components analysis (PCA). The numbers of participants removed in each step are reported in Table S1.

*Imputation*. Imputation after quality control was processed by MaCH and MiniMac with 1000 Genomic Phase 1 Version 3 CEU as the reference datasets. The genotyped data were aligned to the human reference genome of GRCh37/hg19 (Howie et al., 2012; Li et al., 2010). The criteria of MAF were set as larger than 0.01 and the imputation info quality score was set as larger than 0.8. After genetic quality control, we included 304 qualified aMCI participants with qualified 7,747,882 imputed genetic variants in further analysis.

#### 2.2.2 UK Biobank

We used the imputed genomic data (Version 3) made available by UKBB with 487,411 individuals (Bycroft et al., 2018), which was imputed from the Haplotype Reference Consortium reference panel and a merged UK10K and 1000 Genomes phase 3 reference panels. In participants‐level quality control, we applied exclusion filters for participants as follows: (1) participants with a mismatch in reported sex and chromosome X‐imputed sex or with putative sex chromosome aneuploidy; (2) participants with genetic kinship to other participants; (3) excess heterozygosity or missing rates; (4) non‐Caucasian participants; (5) without calculated genetic principal components. In Genetic variants‐level quality control, we applied exclusion filters for Genetic variants as follows: (1) MAF <0.001; (2) imputation info quality score >0.3. A total of 275,988 participants and 13,918,727 genetic variants were finally used in the further analysis.

### 2.3 PRS calculation

PRS is an estimate of an individual's genetic susceptibility to the neuroticism trait. The discovery sample could recognize a certain number of genetic variants associated with neuroticism at a chosen *p* value threshold (*p*_T_). Clumping was applied in the remaining genetic variants to extract the index genetic variants using linkage disequilibrium (LD) of *r* ^2^ >.5 within a 250 kb window. Index genetic variants were selected as risk alleles from each clumped association region and obtained their effect sizes. In the target sample, PRS was calculated for each participant as the sum of the count of risk alleles multiplied by the corresponding effect sizes across these index genetic variants. The effect size of *β* coefficients and reference alleles of genetic variants were obtained from discovery samples of neuroticism GWAS summary statistics in the target sample of ADNI and UKBB, separately.

To identify the *p*_*T*_ value that could construct PRS with the best prediction for the conversion from aMCI to AD, PRSice v2.0 software (http://prsice.info) (Choi & O'Reilly, 2019; Euesden et al., 2015) was used to generate 1000 PRS values for *p*_*T*_ ranging from .001 to 1 with an increment of .001, while controlling for gender, age, sites, and educational years at baseline, the number of *APOE* ε*4*, and the first four PCA components for population stratification. *p*_T_ = 1 indicated that all genetic variants of the discovery sample were included in calculating the PRS in the target sample. By evaluating the predictive abilities, we could obtain the optimal *p*_T_ values for calculating N‐PRS in the target sample. With the optimal *p*_T_, we could obtain the risk alleles, effect sizes of the index genetic variants, and the corresponding N‐PRS for each aMCI patient in the target sample. The N‐PRS was further *z*‐transformed into the further analyses.

In the ADNI, we first constructed N‐PRS_1_ based on the first discovery sample to predict the conversion of aMCI patients in the ADNI. There were 20 outliers of scaled N‐PRS_1_ based on mean ± 2 standard deviation and were further excluded, where 284 aMCI patients were retained in the subsequent analysis. To exclude the technical bias of the discovery neuroticism GWAS sample, we constructed N‐PRS_2_ based on the second discovery sample to replicate the prediction effect of N‐PRS_1_ on aMCI conversion in ADNI.

In the UKBB, to exclude sample overlapping between the discovery and target sample, we constructed N‐PRS based on the second discovery sample in the UKBB as the same process. Finally, the 933 aMCI participants with qualified genetic and follow‐up diagnosis data were used as an independent external validation dataset.

### 2.4 Neuroimaging data process

The aMCI participants were scanned with a standardized MRI protocol developed for ADNI‐1 (Jack et al., 2008). Details about the rationale and development of the standardized MRI datasets had been previously described (Wyman et al., 2013). High‐resolution structural MRI data were acquired at 59 locations using a 1.5 T MRI scanner with a sagittal 3D magnetization prepared rapid acquisition gradient echo sequence (http://adni.loni.ucla.edu). The acquired sites were additionally controlled in the relative neuroimaging analyses.

All structural images were visually checked by two experimenters of radiology. In the 284 participants with qualified genetic data, we removed 6 participants because of poor image quality. Finally, a total of 278 aMCI patients were finally included in the neuroimaging analyses. Briefly, Freesurfer was applied to qualified T1 weighted data to generate the cortical volume (VL), cortical thickness (CT), and surface area (SA) of 130 brain regions (http://surfer.nmr.mgh.harvard.edu/fswiki/FreeSurferMethodsCitation) using the Desikan–Killiany atlas and the Destrieux atlas (Desikan et al., 2006; Reuter et al., 2012), which was provided by the ADNI‐1 (Fischl, 2012; Schwarz et al., 2016).

In the UKBB dataset, among the 933 aMCI patients, only 14 patients have been scanned for MRI data. Thus, we did not perform the neuroimaging‐related analyses in the UKBB because such a small sample size is not sufficient to validate the results in ADNI.

### 2.5 Statistical analysis

#### 2.5.1 Demographic data

We performed statistical analyses using SPSS version 20.0 (SPSS Inc, Chicago, IL, USA) and R version 4.0.5. The descriptive statistics of demographic data in aMCI‐C and aMCI‐S groups at 1‐ and 10‐year follow‐up in ADNI are shown in Table 1. The descriptive statistics of demographic data in aMCI‐C and aMCI‐S groups in UKBB are shown in Table S2.

**TABLE 1:** Demographic of aMCI patients in ADNI.

Demographic variables	1‐Year follow‐up				10‐Year follow‐up			
	aMCI‐S	aMCI‐C	Statistics	*p* Value	aMCI‐S	aMCI‐C	Statistics	*p* Value
Sample size (*n*)	234	44	–	–	116	162	–	–
Males/females (*n*)	152/82	26/18	0.55	.457	76/40	102/60	0.19	.662
Age at baseline (years)	75.55 (7.00)	73.29 (8.01)	1.92	.056	75.93 (6.82)	74.65 (7.44)	1.46	.145
*APOE* ε4 status (*n*)^a^	129/105	29/15	1.75	.185	53/63	105/57	10.08	***.002***
Educational years	15.78 (2.99)	15.34 (2.87)	0.89	.372	15.56 (3.12)	15.81 (2.87)	−0.70	.483

*Note*: Data are shown as mean (SD); *p* values in bold and italic indicate significant differences between aMCI‐S and aMCI‐C groups.

Abbreviations: aMCI‐C, amnestic mild cognitive impairment converted; aMCI‐S, amnestic mild cognitive impairment stable.

^a^ *APOE* ε4 status shows the participants with ε4 carriers (one or two numbers of ε4 allele at the *APOE* locus) and non‐carriers;

#### 2.5.2 Prediction effect of N‐PRS on aMCI conversion

In 278 aMCI participants from the ADNI, we tested the predictive effect of N‐PRS on the conversion of aMCI at 1‐ and 10‐year follow‐up. Independent two‐sample *t*‐test was first applied to investigate the statistical group differences of N‐PRS_1_ between aMCI‐C and aMCI‐S groups. Then, using the N‐PRS_1_ calculated under *p*_*T*_ thresholds as a predictor, the logistic regression was used to predict the conversion risk for aMCI to AD. The permutation test (*p* < .05) was used to correct multiple comparisons. Nagelkerke's pseudo *R* ^2^ was calculated to measure the proportion of variance explained by the N‐PRS_1_ for prediction. For the best‐fitting N‐PRS, odd ratios (ORs) for these variables were calculated using the prediction models. The receiver operating characteristic curve was used to demonstrate the respective N‐PRS_1_ to discriminate between aMCI‐S and aMCI‐C. Cox survival analysis was used to explore the relations between the N‐PRS_1_ and the conversion of aMCI at different time points. To exclude the technical bias of the first discovery neuroticism GWAS sample, we constructed N‐PRS_2_ based on the second discovery sample to replicate the prediction effect of N‐PRS_1_ on aMCI conversion in ADNI. In addition, the predictive effect of N‐PRS on the aMCI conversion would be replicated in an independent external dataset of UKBB. The significant level was set as *p* < .05, indicating the results were considered verifiable.

To exclude the effect of neuroticism‐related mental traits PRS on the conversion of aMCI to AD, we generated PRS for general cognition (GC‐PRS) using GWAS summary statistic data of general cognition derived from UKBB (Davies et al., 2019), PRS for depression (DEP‐PRS) using meta‐analysis GWAS summary statistic data of major depressive disorder derived from Psychiatric Genomics Consortium (Wray et al., 2018), and PRS for anxiety (ANX‐PRS) using meta‐analysis GWAS summary statistic data of primary anxiety disorders derived from Psychiatric Genomics Consortium (Otowa et al., 2016) (Supporting Information Methods). In 278 aMCI patients, we compared the independent prediction effect of N‐PRS_1_, GC‐PRS, DEP‐PRS, and ANX‐PRS on the conversion from aMCI to AD using logistic regression analysis, controlling for the same confounding factors. Also, additionally controlling the GC‐PRS, DEP‐PRS, and ANX‐PRS, we further tested the prediction effect of N‐PRS_1_ on the aMCI conversion. The same process was also applied to N‐PRS_2_.

To directly compare the difference in conversion rate of aMCI among N‐PRS_1_ hierarchical risk groups, we dichotomized the 278 aMCI patients into a low‐N‐PRS_1_ group (*n* = 139) and a high‐N‐PRS_1_ group (*n* = 139). A chi‐square test was used to compare the statistically significant differences in conversion rates of aMCI among the low‐N‐PRS_1_ group and high‐N‐PRS_1_ group. To exclude the bias of arbitrarily dichotomization of the risk group, we also trichotomized the N‐PRS_1_, the bottom third (*n* = 92) was defined as the low‐risk group, the middle third (*n* = 93) as the middle‐risk group, and the upper third (*n* = 93) as the high‐risk group. We also compared differences in the conversion rate of aMCI among the three N‐PRS_1_ hierarchical risk groups. To replicate the statistical difference in the conversion rate of aMCI patients among N‐PRS_1_ hierarchical risk groups, we also compare the conversion rate of aMCI in hierarchical risk groups of N‐PRS_2_ in the ADNI dataset.

#### 2.5.3 Correlations of N‐PRS with brain structural phenotypes

To test the neural mechanisms underlying the correlations of N‐PRS_1_ with aMCI conversion, Pearson correlation analysis was first conducted to test the associations between N‐PRS_1_ and brain structural phenotypes, controlling for age, gender, educational year, sites, *APOE* ε4 and the first four PCA components. Multiple comparisons correction of FDR *q* < .05 were used as the threshold of significance. The statistically significant brain structural phenotypes correlated with N‐PRS_1_ were used for further analyses. Correlation coefficient value (*r* value), explained variance and *p* value would be reported. To replicate the brain structural phenotypes correlated with N‐PRS_1_, the significant brain structural phenotypes would be replicated in the associations of N‐PRS_2_ with aMCI conversion at *p* < .05.

#### 2.5.4 One sample Mendelian randomization analysis

To make potentially causal inferences between N‐PRS_1_, brain structural phenotypes and aMCI conversion, individual‐level one sample Mendelian randomization was further performed using a two‐stage least square method (TSLS) (MR‐TSLS) using *ivreg* R package (https://www.rdocumentation.org/packages/ivreg/versions/0.6-2). In the MR‐TSLS analysis, the N‐PRS_1_ was used as the IV, significant brain structural phenotypes were used as exposure variables and aMCI conversion was used as the binary outcome variable (Bowden et al., 2015; Burgess, 2014; Burgess & Thompson, 2013). The *β* coefficient from the MR‐TSLS analyses can be interpreted as the change in the aMCI conversion per standard deviation increase in the brain structural phenotypes due to the N‐PRS. To replicate the potentially causal chains from N‐PRS_1_ to brain structural phenotypes to aMCI conversion, the significant brain structural phenotypes would be replicated in the causal associations from N‐PRS_2_ to brain structure to aMCI conversion using the MR‐TSLS at *p* < .05.

## 3 RESULTS

### 3.1 Demographics

The *chi‐square* test or independent two‐sample *t*‐test were used to compare the differences in gender, age at baseline, educational years and *APOE* ε4 carriers between the aMCI‐S and the aMCI‐C group at 1‐ and 10‐year follow‐up, separately (Table 1). There were no significant group differences in gender, age at baseline, and educational years between the two groups (*p >* .05). In the aMCI‐C group, the ratio of *APOE* ε4 was significantly higher than those in the aMCI‐S group at 10‐year follow‐up (*χ* ^2^ = 10.08, *p =* .002), though not significant at 1‐year follow‐up.

### 3.2 N‐PRS predict conversion from aMCI to AD at early stage

**FIGURE 1:**
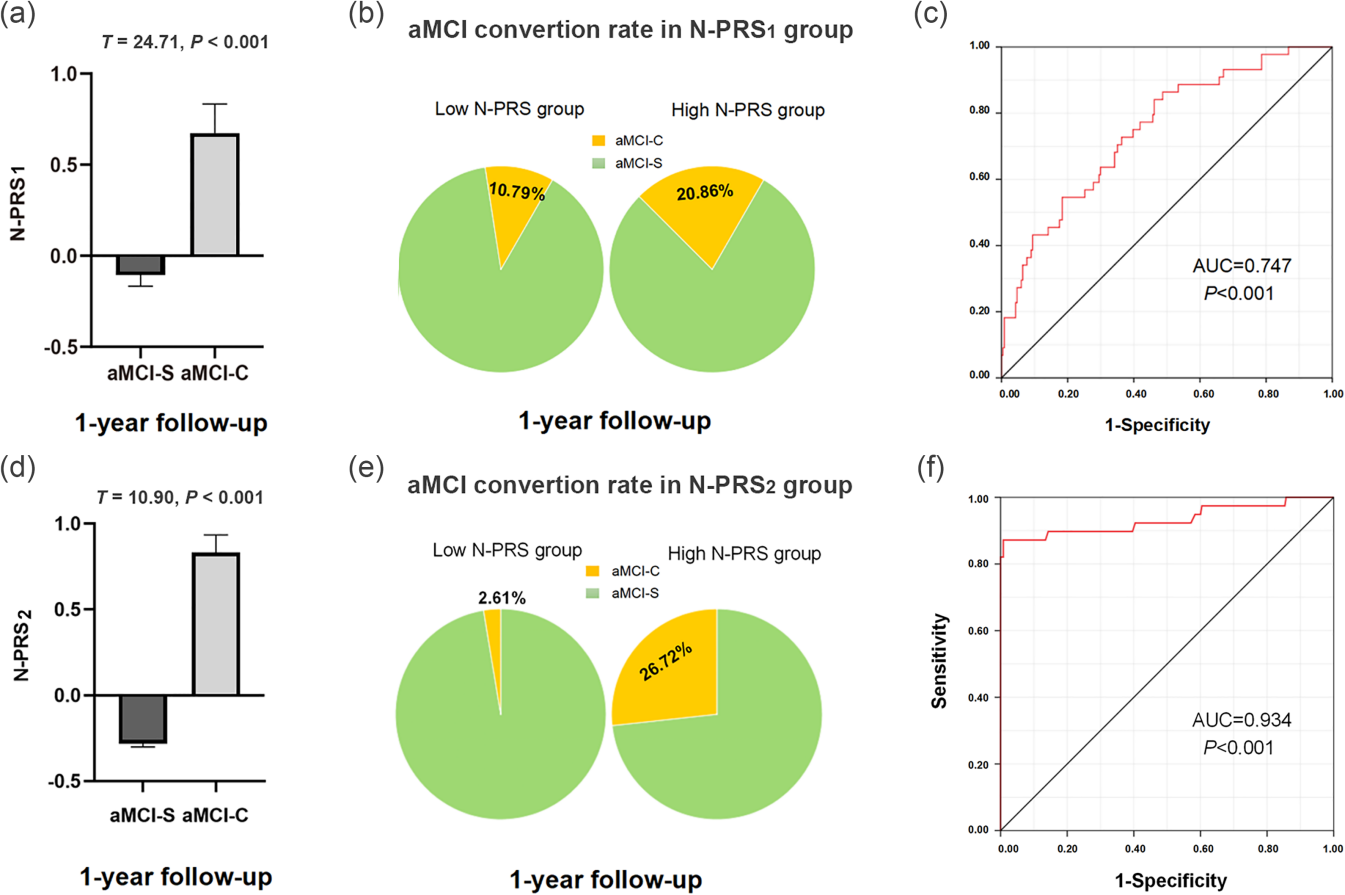
Predictive effect of N‐PRS on conversion of aMCI to AD at 1‐year follow‐up in ADNI. (a, d) Mean and standard deviation of N‐PRS_1_ (a) and N‐PRS_2_ (d) at 1‐year follow‐up in the aMCI‐C and aMCI‐S groups. (b, e) Conversion rates at 1‐year follow‐up for low group (left) and high group (right) of N‐PRS_1_ (b) and N‐PRS_2_ (e). In the high group, aMCI conversion rate (20.86% for N‐PRS_1_, 26.72% for N‐PRS_2_) was significantly higher than those (10.79% for N‐PRS_1_, 2.61% for N‐PRS_2_) in the low group. (c, f) At 1‐year follow‐up, the AUC was 0.747 in the predictive effect of N‐PRS_1_ (c) and 0.934 of N‐PRS_2_ (f) in the ROC curve of logistic regression for the aMCI conversion risk. aMCI‐C, amnestic mild cognitive impairment converted to AD group; aMCI‐S, amnestic mild cognitive impairment stable group; AUC, area under the curve; N‐PRS_1_, cumulative genetic risk for neuroticism based on the first discovery sample in the target ADNI sample; N‐PRS_2_, cumulative genetic risk for neuroticism based on the second discovery sample in the target ADNI sample.

N‐PRS_1_ demonstrated cumulative genetic risk for neuroticism based on the largest and most powerful neuroticism GWAS summary data in the 278 aMCI patients from the ADNI sample. We found that the N‐PRS_1_ in the aMCI‐C group was significantly higher than those in the aMCI‐S group at 1‐year follow‐up (*T* = 24.71, *p <* .001) (Figure 1a), but not at 10‐year follow‐up (*T* = 1.67, *p =* .20). The N‐PRS_1_ was divided into high‐ and low‐risk groups, and there was significant difference in N‐PRS_1_ between the groups (*p* < .001). We calculated the aMCI conversion rate at 1‐year follow‐up in low‐ and high‐N‐PRS_1_ groups, separately. At 1‐year follow‐up, the aMCI conversion rate in the high‐N‐PRS_1_ group (20.86%) was significantly higher than those in the low‐N‐PRS_1_ group (10.79%) (*χ* ^2^ = 5.292, *p* = .021) (Figure 1b). There were also significant differences in the conversion rate of aMCI among the three hierarchical N‐PRS_1_ groups (*p* = 1.05e−4). To test the prediction effect of N‐PRS_1_ on the conversion from aMCI to AD, we applied logistic regression using N‐PRS_1_ as an independent variable, controlling for the confounding variables. At 1‐year follow‐up, we found each unit increase in N‐PRS_1_ demonstrated a 0.80‐fold increment of the aMCI conversion risk (ORs = 2.23, *p* = 2.6e−5), where the area under the curve (AUC) was 0.747 (Figure 1c) in the logistic regression analysis. The Cox survival analyses showed the correlations of N‐PRS_1_ with the conversion from aMCI to AD at different time points and the cumulative risk proportion for aMCI‐C at any given 1‐year follow‐up period (*p* < .001). However, at the 10‐year follow‐up, the N‐PRS_1_ could not predict the conversion of aMCI to AD (ORs = 0.848, *p* = .203). These results showed that N‐PRS_1_ could sensitively predict the conversion of aMCI to AD at an earlier stage.

To exclude the technical bias of the first discovery neuroticism GWAS sample, we constructed N‐PRS_2_ based on the second discovery sample to replicate the prediction effect of N‐PRS_1_ on aMCI conversion in ADNI. We additionally validated the results using N‐PRS_2_ as predictors in the conversion of 278 aMCI patients from ADNI. We found that the N‐PRS_2_ in the aMCI‐C group was still significantly higher than those in the aMCI‐S group at 1‐year follow‐up (*T* = 10.90, *p <* .001) (Figure 1d). At 1‐year follow‐up, aMCI conversion rate in the high‐N‐PRS_2_ group (26.72%) was significantly higher than those in the low‐N‐PRS_2_ group (2.61%) (*χ* ^2^ = 34.61, *p <* .001) (Figure 1e). There were also significant differences in the conversion rate of aMCI among the three hierarchical N‐PRS_2_ groups (*p* = 1.40e−5). The prediction effect of N‐PRS_2_ on the aMCI conversion at 1‐year follow‐up could be well replicated in the logistic regression (*p* = 5.04e−9) and Cox survival analysis (*p* < .001) using N‐PRS_2_ as predictors. We found that each unit increase in N‐PRS_2_ demonstrated a 1.74‐fold increment of the aMCI conversion risk (ORs = 5.67, *p* = 5.04e−9), where the AUC was 0.934 (Figure 1f).

To exclude the effect of neuroticism‐related mental traits PRS on the conversion of aMCI to AD at 1‐year follow‐up, we generated GC‐PRS, DEP‐PRS, and ANX‐PRS in 278 aMCI patients (Methods). In 278 aMCI patients, we compared the prediction effect of four PRSs on the conversion from aMCI to AD at 1‐year follow‐up using logistic regression analysis, controlling for the same confounding factors. We found that only N‐PRS_1_ (ORs = 2.23, *p* = 2.60e−5) could predict the conversion from aMCI to AD, while not for GC‐PRS (OR = 1.305, *p* = .155), DEP‐PRS (ORs = 0.785, *p* = .169), and ANX‐PRS (ORs = 0.728, *p* = .095) (Figure S1a). After additionally controlling for the GC‐PRS, DEP‐PRS, and ANX‐PRS in the prediction of the N‐PRS_1_, we found that N‐PRS_1_ still could predict the conversion of aMCI to AD (*p* = 3.40e−5) (Figure S1b). This result could be well replicated in the prediction effect of N‐PRS_2_ on the aMCI conversion while controlling these three mental traits PRSs (*p* = 1.00e−8) (Figure S1c).

To replicate the predictive effect of N‐PRS on the conversion of aMCI in ADNI, we validated the predictive effect of N‐PRS in the conversion of qualified 933 aMCI patients from an independent dataset of UKBB. In the aMCI‐C group (*n* = 113), we found that the N‐PRS was significantly higher than those in the aMCI‐S group (*T* = 2.2485, *p* = .026) (Figure S2). The prediction effect of N‐PRS on the aMCI conversion could be well replicated in the logistic regression using N‐PRS as predictors in the UKBB (*p* = .007). We found that each unit increase in N‐PRS demonstrated a 0.3‐fold increment of the aMCI conversion risk (ORs = 1.35, *p* = .007). These results showed that N‐PRS could independently and robustly predict the conversion of aMCI to AD at an early stage.

### 3.3 Correlations between N‐PRS and brain structural phenotypes

**FIGURE 2:**
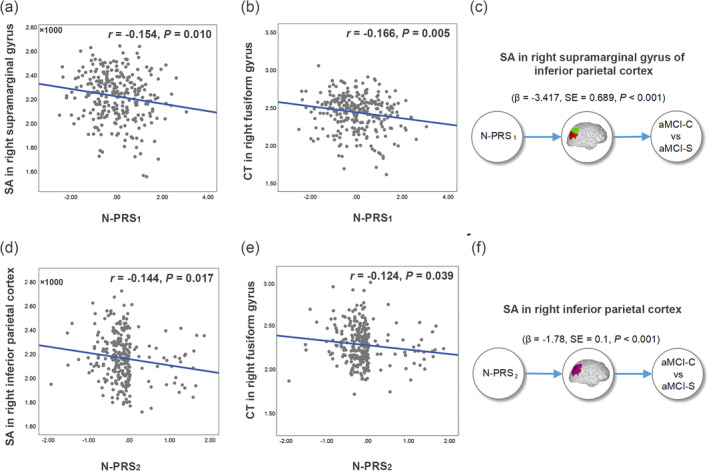
Casual associations between N‐PRS, brain structure, and aMCI conversion. (a, b) N‐PRS_1_ was significantly negatively correlated with right supramarginal of inferior parietal surface area (a) and right fusiform gyrus cortical thickness (b). (c) Causal associations between N‐PRS_1_, right supramarginal of inferior parietal surface area, and aMCI conversion using one sample MR‐TSLS analyses. (d, e) N‐PRS_2_ was significantly negatively correlated with right inferior parietal surface area (d) and right fusiform gyrus cortical thickness (e). (f) Causal associations between N‐PRS_2_, right inferior parietal surface area, and aMCI conversion using one sample MR‐TSLS analyses. Green color represents the right supramarginal gyrus. aMCI‐C, amnestic mild cognitive impairment converted; aMCI‐S, amnestic mild cognitive impairment stable; MR‐TSLS, one sample Mendelian randomization two‐stage least square method; N‐PRS, neuroticism polygenic risk score.

To test the neural mechanism underlying the associations between N‐PRS_1_ and aMCI conversion, *Pearson* correlation analyses were first used to explore the associations between the N‐PRS_1_ and brain structural phenotypes in 278 aMCI patients. We found that N‐PRS_1_ was significantly negatively correlated with cortical thickness in bilateral postcentral gyrus (right: *r* = −.195, *p* = .001; left: *r* = −.172, *p* = .004), bilateral cuneus (right: *r* = −.157, *p* = .009; left: *r* = −.194, *p* = .001), left precentral gyrus (*r* = −.173, *p* = .004), right paracentral gyrus (*r* = −.173, *p* = .004), right lingual gyrus (*r* = −.170, *p* = .004), right fusiform (*r* = −.166, *p* = .005), left superior parietal gyrus (*r* = −.159, *p* = .008), right rostral middle frontal gyrus (*r* = −.158, *p* = .008), bilateral precuneus (right: *r* = −.155, *p* = .010; left: *r* = −.152, *p* = .010), right superior frontal gyrus (*r* = −.147, *p* = .014), bilateral middle temporal gyrus (right: *r* = −.141, *p* = .018; left: *r* = −.146, *p* = .015), and left medial orbitofrontal gyrus (*r* = −.142, *p* = .018), as well as surface area in right supramarginal gyrus of inferior parietal cortex (IPC) (*r* = −.154, *p* = .010) (Figure 2). Also, N‐PRS_1_ was found significantly positively correlated with surface area in right transverse temporal gyrus (*r* = .163, *p* = .006), left entorhinal gyrus (*r* = .145, *p* = .015), right parahippocampal gyrus (*r* = .142, *p* = .018) and left posterior cingulate gyrus (*r* = .141, *p* = .019) (Figure 2). Among these brain regions' structure, two brain regions' structure could be well replicated in the associations with N‐PRS_2_, including the surface area in the right supramarginal gyrus of IPC (*r* = −.144, *p* = .017) and cortical thickness in the right fusiform gyrus (*r* = −.124, *p* = .039) in ADNI. These results indicate that the genetic cumulative risk of neuroticism is negatively associated with the right inferior parietal and lateral occipitotemporal structure, which were key brain regions for cognitive ability impairment in AD patients (Chochon et al., 1999; Eknoyan et al., 2012; Golaszewski et al., 2021; Keilp et al., 1996).

### 3.4 Causal associations between N‐PRS, brain structure, and aMCI conversion

Based on the two replicated brain structures, to make potentially causal inferences between N‐PRS, brain structure phenotypes and aMCI conversion, one sample MR‐TSLS analysis was applied in 278 aMCI patients. In one sample MR‐TSLS regression analysis, we found a significant potentially causal association between higher N‐PRS_1_, lower surface area in the right supramarginal gyrus of IPC and higher conversion risk of aMCI patients at 1‐year follow‐up (*β* = −3.42, SE = 0.69, *p* < .001), while not for cortical thickness in right fusiform gyrus (*p* = .68). These results could be well replicated when the N‐PRS_2_ was regarded as IV. We replicated the potential causal association from higher N‐PRS_2_, lower surface area in the right supramarginal gyrus and higher conversion risk of aMCI patients at 1‐year follow‐up in ADNI (*β* = −1.78, SE = 0.10, *p* < .001). The right supramarginal gyrus is one of the three parts of the IPC. These results demonstrated that N‐PRS impaired the right inferior parietal structure, a key brain region associated with psychosis, which ultimately leads to the conversion risk of aMCI to AD. These results provided the potential neural mechanism underlying the predictive effect of N‐PRS on aMCI conversion.

## 4 DISCUSSION

In the present study, we demonstrated that the N‐PRS could predict the conversion of aMCI to AD, which were well replicated in internal dataset as well as an independent external dataset of 933 aMCI patients from UKBB. We further explored the neural mechanisms underlying the predictive effect of N‐PRS. We found that the right inferior parietal structure has a causal role in the predictive effect of N‐PRS on the conversion of aMCI to AD. The higher the risk of N‐PRS, the higher the degree of right inferior parietal lobule atrophy and the higher the aMCI‐AD conversion rate.

AD is a polygenic irreversible neurodegenerative disease. Although aducanumab has been considered a therapeutic drug for AD, controversy remains over the efficacy of the treatment (Selkoe, 2021; Servick, 2021). There is no very effective treatment in the late stages of AD currently, thus early diagnosis and treatment are the best way to improve the prognosis. aMCI is the pre‐pathological state of AD (Gauthier et al., 2006) and approximately one of third of aMCI patients will develop AD (Chaudhury et al., 2019). Therefore, predicting conversion from aMCI to AD is clinically important for early intervention.

Increasing evidence showed that neuroticism shared a partial genetic association with AD (Nagel et al., 2018). N‐PRS has better predictive validity for the development of AD, with high N‐PRS showing accelerated AD development compared with low N‐PRS (Terracciano et al., 2014). With the development of second‐generation genomic sequencing technology, more accurate genomic data can be obtained for more aMCI patients, providing more possibilities for constructing N‐PRS. In clinical practice, the N‐PRS can be employed to distinguish aMCI patients with higher conversion risk of AD, and appropriate interventions for these aMCI patients can prevent or delay their progression to AD (Li et al., 2022). We found that higher N‐PRS was associated with increased conversion rate of aMCI. This is consistent with previous findings that PRS for personality was associated with the conversion of aMCI (Li et al., 2022; Lupton et al., 2021). While during a long period of 10‐year follow‐up, the N‐PRS could not predict the conversion of aMCI to AD in the present study. One explanation is that neuroticism is a temporary trait disposition to experience negative life affects (Costa & McCrae, 1992), and might not be inessive to the long‐term prediction of aMCI. Also, with the increasing follow‐up time, there are more and more confounding factors, such as medication status, affecting the conversion of aMCI to AD, leaving the predictive effect of N‐PRS on the conversion of aMCI insensitive.

The neuroticism personality showed genetic susceptibility to AD by exacerbating cognitive impairment (Zufferey et al., 2017), disturbing emotional fragility (Danielsdottir et al., 2019) and sleep difficulties (Stephan et al., 2020), which in turn makes people more susceptible to various cardiovascular diseases (Lee et al., 2022), all of which are high‐risk factors for AD (Andrews et al., 2021; Korologou‐Linden et al., 2019; Li et al., 2021; Stephan et al., 2018). Furthermore, neuroticism is an important risk factor for many psychiatric disorders, such as major depressive disorder and schizophrenia (Brainstorm Consortium et al., 2018; Genetics of Personality Consortium et al., 2015). The genetic associations between neuroticism personality and psychiatric disorders can affect the development of AD (DeMichele‐Sweet et al., 2018; Kusters et al., 2020; Xu et al., 2018). Thus, the cumulative genetic risk of neuroticism could consider as a predictive factor for the aMCI conversion. Furthermore, we only found that N‐PRS could predict the aMCI conversion at an earlier stage, naming 1‐year follow‐up. It indicated that neuroticism‐related genes may modulate modified and reversible gene elements and functions underlying the aMCI conversion (Dar‐Nimrod et al., 2012; De Jager et al., 2021; Yoneda et al., 2020) at an earlier stage.

The present study first provided correlations between N‐PRS and brain structural phenotypes. The neuroticism has been recognized as negatively correlated with inferior parietal structure (Opel et al., 2020). The polygenetic architecture of neuroticism has been proposed as an important cause for the reduction of inferior parietal structure. For example, N‐PRS was found to be significantly associated with decreased inferior parietal surface area in independent discovery and replication datasets (Opel et al., 2020), which was consistent with the negative correlation of N‐PRS with IPC surface area in our study. The IPC plays an important role in episodic memory and is one of the specific neuroimaging markers in predicting the conversion of aMCI to AD (Chao et al., 2010; Sedaghat et al., 2010). For example, compared with stable aMCI diagnosis status patients, aMCI converters showed significantly decreased inferior parietal structure and corresponding impaired executive function (Rainville et al., 2012). In addition, in the connectivity of IPC, compared with low neurotic individuals, high neurotic individuals exhibited overall weaker functional connections of the IPC in executive control network, default mode network and salience network (Servaas et al., 2015). These functional networks are precisely the most vulnerable network of being impairment in aMCI and AD patients (Chand et al., 2017). Thus, the additional burden to the IPC and its related functional network by neuroticism genetic risk may facilitate the conversion from aMCI to AD.

Also, one sample Mendelian randomization analyses confirmed that the right IPC surface area played a causal role in the predictive effect of N‐PRS on the conversion of aMCI to AD. Atrophic brain structural phenotypes are prominently early pathological features of AD (Yang et al., 2012) as well as conversion from aMCI to AD (Brueggen et al., 2015). The additional burden of neuroticism polygenetic architecture on the brain structural phenotypes may exacerbate cortical atrophy and facilitate the conversion of aMCI to AD. Thus, the IPC atrophy may explain the increased predictive value of N‐PRS for the conversion of aMCI to AD. These findings suggested that the right inferior parietal atrophy has a reliable internal association between neuroticism polygenetic architecture and aMCI conversion. Our result demonstrated the causal chain from N‐PRS to the right‐side IPC surface area and aMCI conversion, while not the left side. The brain regional lateralization effect of N‐PRS on IPC structure was supported by the previous result (Gao et al., 2013; Ueda et al., 2018).

Regarding potential limitations of the study, investigating the effect of N‐PRS on the conversion of aMCI to AD, besides controlling for age, gender, and *APOE* ε4, requires consideration of other risk factors that influence AD progression, such as whether the patient has other comorbid neuropsychiatric diseases. In addition, the identified genetic loci for neuroticism only explained limited variation for the trait. The underlying environmental effect and gene × environment interaction effect of neuroticism may play the other important role underlying the conversion of aMCI to AD. Thus, further research is needed in the future to remedy these limitations.

In conclusion, the present work discovered and replicated that N‐PRS could predict the conversion of aMCI to AD. In the early stages of follow‐up, N‐PRS showed excellent predictive performance. There were specific brain regions, including the right inferior parietal surface area, negatively associated with N‐PRS. The right inferior parietal structure had a causal effect of N‐PRS on the conversion of aMCI to AD. A higher N‐PRS resulted in a higher degree of atrophy of the right inferior parietal structure, which finally led to a higher conversion of aMCI to AD. This study provided the causal neural mechanisms underlying the predictive effect of the cumulative genetic risk of neuroticism on the conversion of aMCI to AD.

## CONFLICT OF INTEREST STATEMENT

The authors declare no conflict of interest.

## Supporting information


**Data S1.** Supporting Information.

## Data Availability

The data that support the findings of this study are available in Pubmed at https://pubmed.ncbi.nlm.nih.gov/. These data were derived from the following resources available in the public domain: Genetics of Personality Consortium, https://tweelingenregister.vu.nl/gpc; Alzheimer's Disease Neuroimaging Initiative, http://www.adni-info.org; UK Biobank dataset, https://www.ukbiobank.ac.uk/; GWAS for for neuroticism (Nagel et al., 2018), https://www.ebi.ac.uk/gwas/publications/29942085.
