# Supplementary material for: Neuroticism polygenic risk predicts conversion from mild cognitive impairment to Alzheimer's disease by impairing inferior parietal surface area
Source: Hum Brain Mapp. 2024 May 15;45(7):e26709. doi: 10.1002/hbm.26709 (PMC11094517; doi:10.1002/hbm.26709)
Supplement: Supplementary file 1 — Data S1. Supporting Information. [file HBM-45-e26709-s001.docx]

Supplementary Materials

## Materials and Methods

### Discovery sample for GC-PRS, DEP-PRS, and ANX-PRS

In calculating the PRS for general cognition (GC-PRS), PRS for depression (DEP-PRS), PRS for anxiety (ANX-PRS), we used the GWAS summary statistic data of general cognition derived from UK Biobank, major depressive disorder and primary anxiety disorders derived from Psychiatric Genomics Consortium.

GWAS summary statistic data of general cognition derived from UK Biobank (Davies et al., 2018). The UK Biobank is a large-scale biomedical database and research resource that contains de-identified genetic, lifestyle, and health information, as well as biological samples from 500,000 UK participants (<https://www.ukbiobank.ac.uk/>). Between 100,352 and 468,534 participants aged 38–73 years from UK Biobank completed at least one of seven self-administered cognitive functioning tests: prospective memory (PM), pairs matching (Pairs), fluid intelligence (FI), reaction time (RT), symbol digit substitution, trail making A and B to assessment general cognition. Up to 26,005 participants completed at least one of two follow-up assessments of PM, Pairs, FI and RT (Cornelis et al., 2019).

GWAS summary statistic data of major depressive disorder and primary anxiety disorders derived from Psychiatric Genomics Consortium (Otowa et al., 2016; Wray et al., 2018). The Psychiatric Genomics Consortium (PGC) is a collaborative research effort that focuses on the genetic basis of psychiatric disorders, which consortium aims to facilitate large-scale studies that involve the analysis of genomic data to unravel the complex genetic architecture of psychiatric conditions (<https://pgc.unc.edu/about-us/>). PGC of major depressive disorder conducted a genome-wide association meta-analysis based in 135,458 cases and 344,901 controls and identified 44 independent and significant loci with major depressive disorder. PGC of primary anxiety disorders is to provide further insight into the pathophysiology and neurobiological underpinnings of anxiety disorders through the identification of genetic risk variants, leading to the development of novel treatments or biomarkers, which include GWAS in (i) categorical case-control samples (panic disorder, agoraphobia, generalized anxiety disorder, social phobia and specific phobia), (ii) population based samples with dimensional anxiety measures, and (iii) samples with information on response to treatment for anxiety disorders.

### UKBB subset dataset

To investigate the conversion of aMCI to AD, we defined a subset dataset based on the ICD-10 diagnostic code in filed ID 41270 from the UKBB dataset. In this case, ICD-10 codes F06.7 and F00.0, F00.1, F00.2, F00.9, G30.0, G30.1, G30.8, and G30.9 were used to designate MCI and AD, respectively. When participants possess both the diagnostic codes for MCI and AD, we classified them as aMCI-C. If they only have the code F06.7, we classified them as aMCI-S. If they just have the code AD, we classified them as AD. Lastly, this UKBB subset dataset comprising 929 aMCI-S, 136 aMCI-C, and 2908 AD is available.

We used the aMCI-S and aMCI-C in the subset dataset for analysis to investigate the conversion of aMCI. Ultimately, 933 participants were included in the study following quality control, as the Method section demonstrates. Of these 933 participants, we were able to locate the participants having MRI data, and only 14 individuals from aMCI-S were discovered. As a result, we did not validate any analysis related to the brain. We also did not classify the MCI2AD into 1-year and 10-year follow-up, because no related information is available.

## Results


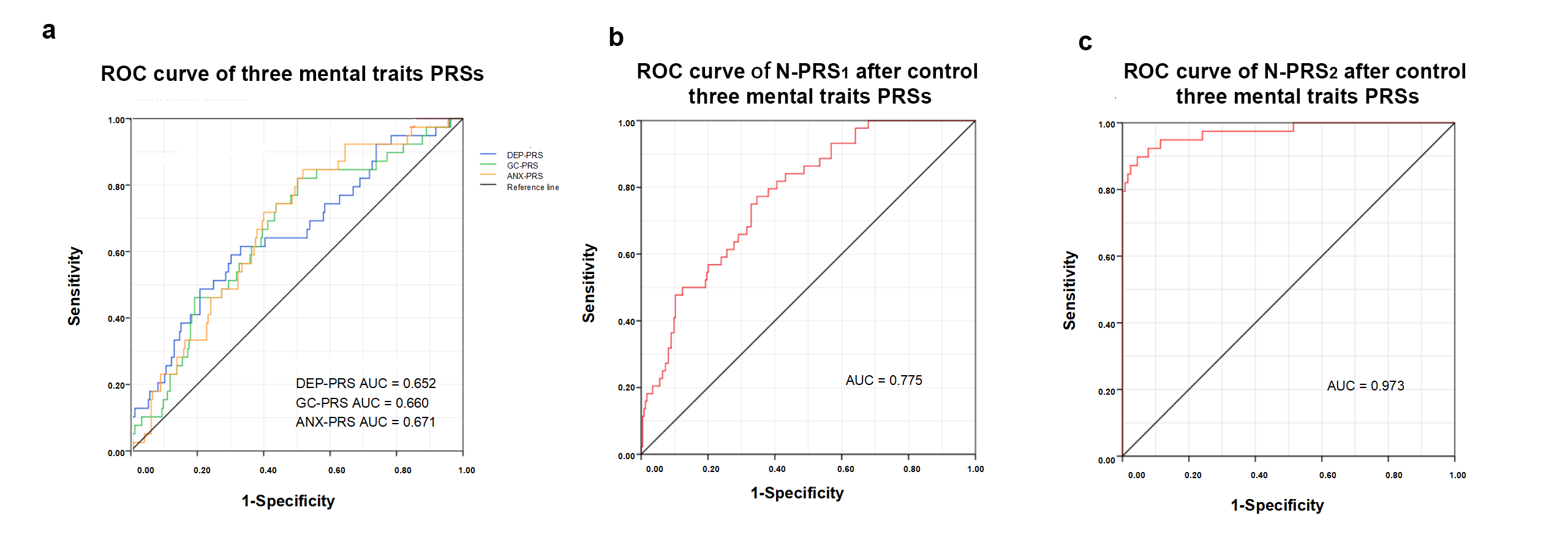


### *Supplementary Figure 1. Prediction effect of N-PRS and other three mental traits PRS on the conversion of aMCI to AD at 1-year follow-up.*

*a. Prediction effect of three mental traits PRS on aMCI conversion. b. Prediction effect of N-PRS_1_ on aMCI conversion while controlling for other three mental traits PRS. c. Prediction effect of N-PRS_2_ on aMCI conversion while controlling for other three mental traits PRS. Abbreviation: N-PRS, neuroticism polygenic risk score; DEP-PRS, depression polygenic risk score; GC-PRS, general cognition polygenic risk score; ANX-PRS, anxiety polygenic risk score.*


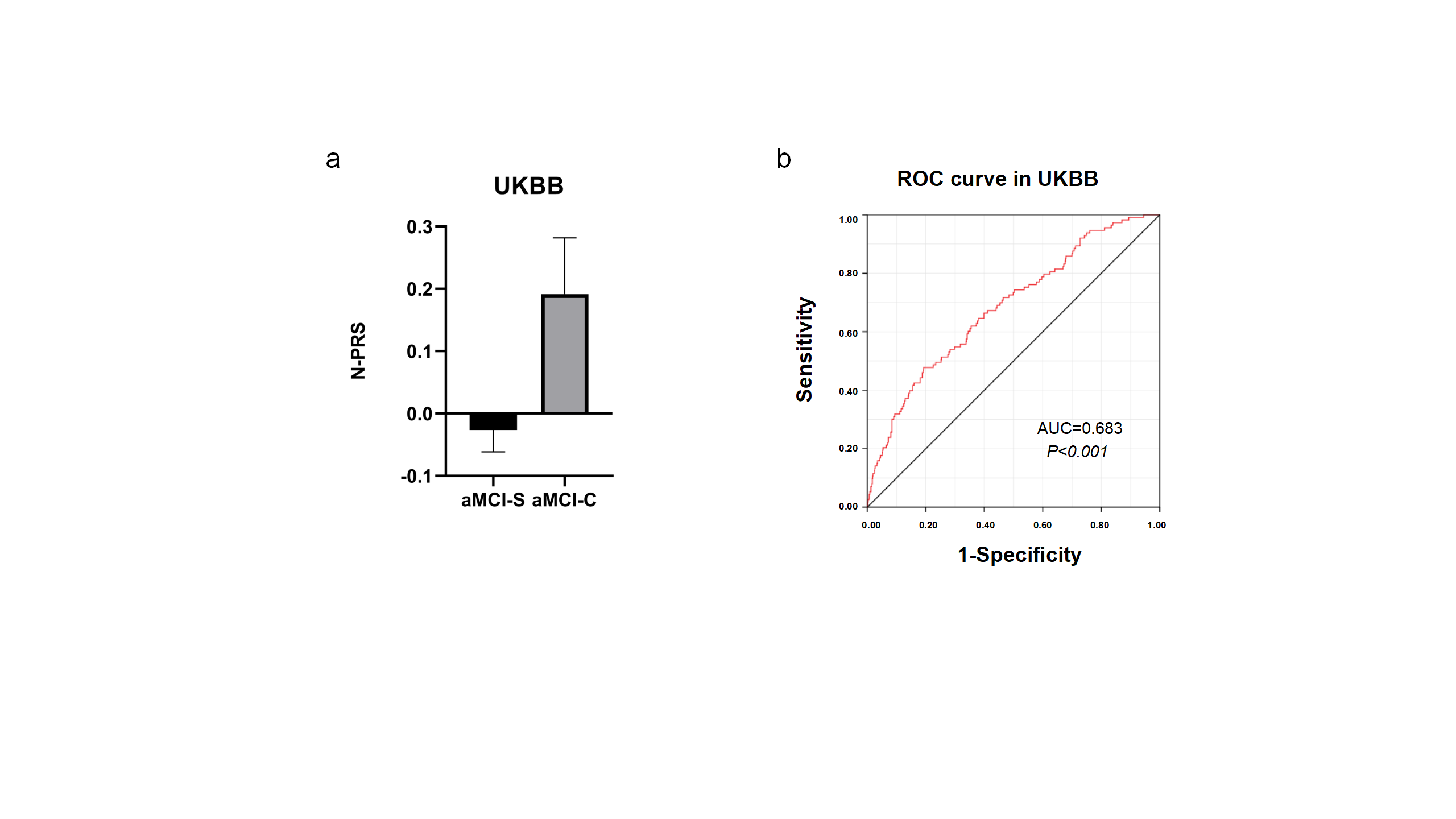


### *Supplementary Figure 2. Predictive effect of N-PRS for conversion of aMCI to AD in UKBB.*

*a. Mean and standard deviation of N-PRS in the aMCI-C and aMCI-S groups. b. In UKBB, the AUC was 0.683 in the ROC curve of logistic regression of N-PRS for the aMCI conversion risk.*

### Supplementary Table 1. Quality control of aMCI patients in ADNI-1.

| Reasons for exclusion | Excluded numbers | Numbers of participants |
| --- | --- | --- |
| **Demographic data** |  |  |
| 1. The total number of aMCI patients |  | 398 |
| 2. Only with the baseline data | 14 | 384 |
| 3. With a normal diagnosis at the follow-up | 9 | 375 |
| 4. Without genotyping data | 9 | 366 |
| **Genetic data process** |  |  |
| 1. Gender inconsistence | 1 | 365 |
| 2. European population outliers | 30 | 335 |
| 3. Poor imputation quality | 31 | 304 |
| **N-PRS calculation** |  |  |
| 1. outliers ($\pm$2 standard deviation) | 20 | 284 |
| **Neuroimaging data process** |  |  |
| 1. Poor image quality | 6 | 278 |
| **Finally included participants** |  | 278 |

*Abbreviation: ADNI, Alzheimer's Disease Neuroimaging Initiative; aMCI, amnestic mild cognitive impairment; MRI, Magnetic Resonance Imaging; N-PRS, neuroticism polygenic risk score.*

### Supplementary Table 2. Demographic of aMCI patients in UKBB

| Demographic variables | aMCI-S | aMCI-C | Statistics | *P* |
| --- | --- | --- | --- | --- |
| Sample size (n) | 820 | 113 | - | - |
| Males/Females (n) | 460/360 | 56/57 | 1.72 | 0.190 |
| Age at baseline (years) | 62.48 (6.20) | 64.87(3.96) | -5.54 | ***9.68e-8*** |
| *APOE* ε4 status (n)* | 413/407 | 40/73 | 8.91 | ***0.003*** |
| Educational years | 16.42 (2.42) | 16.11(1.74) | 1.70 | 0.091 |

*Data are shown as mean (SD); aMCI-S, amnestic mild cognitive impairment stable; aMCI-C, amnestic mild cognitive impairment converted.*

** APOE ε4 status shows the participants with ε4 carriers (one or two numbers of ε4 allele at the APOE locus) and non-carriers;*

*P-values in bold and italic indicates significant differences between aMCI-S and aMCI-C groups.*

**Reference**

Cornelis, M.C., Wang, Y., Holland, T., Agarwal, P., Weintraub, S., and Morris, M.C. (2019). Age and cognitive decline in the UK Biobank. PLoS One *14*, e0213948.

Davies, G., Lam, M., Harris, S.E., Trampush, J.W., Luciano, M., Hill, W.D., Hagenaars, S.P., Ritchie, S.J., Marioni, R.E., Fawns-Ritchie, C.*, et al.* (2018). Study of 300,486 individuals identifies 148 independent genetic loci influencing general cognitive function. Nat Commun *9*, 2098.

Otowa, T., Hek, K., Lee, M., Byrne, E.M., Mirza, S.S., Nivard, M.G., Bigdeli, T., Aggen, S.H., Adkins, D., Wolen, A.*, et al.* (2016). Meta-analysis of genome-wide association studies of anxiety disorders. Mol Psychiatry *21*, 1391-1399.

Wray, N.R., Ripke, S., Mattheisen, M., Trzaskowski, M., Byrne, E.M., Abdellaoui, A., Adams, M.J., Agerbo, E., Air, T.M., Andlauer, T.M.F.*, et al.* (2018). Genome-wide association analyses identify 44 risk variants and refine the genetic architecture of major depression. Nat Genet *50*, 668-681.
